# Do natural ingredients in a dentifrice contribute to prevention of plaque and gingivitis?

**DOI:** 10.1111/idh.12517

**Published:** 2021-07-19

**Authors:** Cees Valkenburg, N. A. Martijn Rosema, Nienke L. Hennequin‐Hoenderdos, Paula A. Versteeg, Dagmar Else Slot, G. A. (Fridus) Van der Weijden

**Affiliations:** ^1^ Department of Periodontology Academic Centre for Dentistry Amsterdam (ACTA) a joint venture between the Faculty of Dentistry of the University of Amsterdam and the Faculty of Dentistry of the Vrije Universiteit Amsterdam Amsterdam The Netherlands

**Keywords:** clinical trial, dental plaque, dentifrice, gingival bleeding, gingivitis, natural ingredients

## Abstract

**Objective:**

To test the effectiveness of a dentifrice containing the turmeric and licorice extract compared to a control for preventing plaque and gingivitis over a four‐month period.

**Material and methods:**

Ninety (non‐dental) participants with moderate gingival inflammation (≥ 40%) were selected. The triple blind study consisted of two phases, namely at first a 3‐week pre‐experimental phase of using an oxygenating and chlorhexidine (CHX) mouthrinse. Secondly, a 4‐month experimental period in which participants were randomly assigned to a test or control group. All were instructed to brush their teeth twice daily for 2 minutes with their assigned dentifrice. Gingival bleeding (BI), plaque (PI) and gingivitis (GI) were assessed.

**Results:**

Eighty participants completed the protocol. At the first assessment in the pre‐experimental phase, the mean scores of all indices showed no differences for the two groups. At the second session, the values of all three parameters had decreased significantly (*p *< 0.001). At the last session, the BI values were 0.52(0.25) for the test group and 0.56(0.25) for the control, the mean GI was 0.27(0.17) for the test group and 0.31(0.16) for the control, and for PI the scores were 1.89(0.46) for the test group and 1.98(0.43) for the control group. Statistical comparison of the scores for the two groups at each stage of the study showed no significant difference for any of the parameters.

**Conclusion:**

Within the limits of the current study design, dentifrice formulation and concentration of turmeric/licorice extracts, the results show that the adjuvant effect of the natural ingredients in the test dentifrice was not evident on clinical parameters of gingivitis and plaque.

## 1 INTRODUCTION

Early morphological and histopathological research showed that an intimate spatial relationship existed between dental plaque and the gingiva and periodontal tissues.^1^ Subsequent studies have provided confirming evidence that optimal control of supragingival plaque is a prerequisite for periodontal health.^1^, ^2^, ^3^ Although it is generally recognized that mechanical cleaning is potentially useful for controlling of supragingival plaque, the expectation that each person should maintain a high standard appears to be beyond most people's capabilities. Few people can sustain the dedication required to consistently perform a suitable daily tooth‐cleaning ritual.^4^

The use of dentifrice and a toothbrush is an integral part of most oral hygiene regimes.^5^ The widespread use of fluoride in dentifrices and the decreased prevalence of caries indicate that therapeutic agents can successfully be incorporated into dentifrice formulations, with no extra effort required by the user.^6^

The additional daily use of anti‐inflammatory compounds may be beneficial in oral care products to prevent the development of gingivitis or periodontitis.^7^ Various medicinal herbs have been used for centuries in traditional medicine,^8^ and there have been many reports about the use of traditional plants and natural products for treating oral diseases.^9^ Among these herbs are turmeric root and licorice. Beneficial actions attributed to turmeric are analgesic, antibacterial, anti‐inflammatory, anti‐tumour, anti‐allergic, antioxidant and astringent.^10^, ^11^ In vitro and vivo studies have indicated the possible potential of licorice and its bioactive constituents for the management of oral diseases.^12^, ^13^ Licorice has been reported to inhibit plaque formation^14^ and to inhibit anticariogenic properties.^15^, ^16^

Dentifrice manufactures are looking for additives that can further enhance the effectiveness of their products. At the same time, consumers are seeking oral hygiene products with natural as part of a healthier lifestyle.^8^, ^17^ These changes, along with marketing of herbal products, have led to increased use of natural compounds in foods, cosmetics and pharmaceutical products.^8^ However, ‘natural’ products are not undoubtedly somehow safer, better, or healthier. With regard to combining herbal products and consumer and marketing trends, it was of interest to explore whether the combination of turmeric and licorice extracts in dentifrice may provide a promising oral care product to prevent gingivitis and periodontitis.

A clinical trial was performed to evaluate the clinical efficacy of these two compounds in an experimental dentifrice formulation. In order not to change the brushing habits of participants and to achieve an improvement in gingival health in a short period of time to a level from which deterioration can be measured, rinsing with antimicrobial in a pre‐experimental phase was chosen as a research model.^18^, ^19^, ^20^The aim of the present study was to evaluate the potential of a dentifrice with natural ingredients to inhibit gingivitis development over the subsequent four months in healthy participants.

## 2 MATERIALS AND METHODS

The recommendations for strengthening the reporting were followed, as suggested by the guidelines outlined in Consolidated Standards of Reporting Trials (CONSORT)^21^ and the checklist of the Template for Intervention Description and Replication (TIDieR) were used.^22^

### 2.1 Ethical procedures

This study followed the Good Clinical Practice (CPMP/ICH/135/95) guidelines, in agreement with the ethical principles of the Declaration of Helsinki (October 2013, Brazil) and in accordance with the Medical Research Involving Human Subjects Act (WMO) and applicable local regulations. The study was approved by the medical ethical committee at Amsterdam Medical Centre (MEC 07/021) and was registered at the Dutch Trial Register (NL1170). The study was conducted at the Department of Periodontology Academic Centre for Dentistry of Amsterdam (ACTA), in the Netherlands.

Before enrolment, all volunteers were provided with verbal and written information regarding the aim, rationale and duration of the study. The investigator explained the details of the trial and the potential risks involved. Prior to the study, an informed consent form was signed by all eligible participants who agreed to participate.

### 2.2 Sample size

To detect a 0.19 difference in scores for bleeding on marginal probing scores between two groups, at an 80% power level with an alpha of 0.05, a sample size of 40 was required. This number was calculated using a standard deviation (SD) of 0.31 based on earlier research.^4^, ^23^, ^24^, ^25^ To allow for dropouts, a sample size of ≥ 45 participants per group was chosen. This study design was also able to discern a difference in plaque scores between two groups of 0.26, with an expected standard deviation of 0.43, an alpha of 0.05 and power of 80%.

### 2.3 Recruitment and inclusion

A total of 90 healthy participants were enrolled. They were non‐dentistry students who had moderate gingivitis and fitted the inclusion criteria; they were recruited from universities and colleges in and around Amsterdam. They were informed about the study in a recruitment letter and at the first appointment and were given a written explanation of the background of the study, its objectives and their involvement. Participants agreed to participate in the study. They were numbered consecutively according to their arrival in the study.

The participants had been screened by a dental hygienist (NAMR). To qualify for inclusion, the participants were required to be 18 years or older, systemically healthy and non‐smokers. They were also required to have at least five teeth per quadrant and to display moderate gingivitis (≥ 40% bleeding on marginal probing). Excluded were those who presented with an orthodontic appliance or a removable (partial) denture, any pathological alterations of the oral mucosa, or overt caries. Also excluded were those:

‐who were pregnant or breastfeeding;
‐with any relevant allergies or on relevant medications;
‐who were participating in professional dental cleaning during the study period or who had participated in a clinical study within the previous 30 days;
‐who were having concomitant therapy;
‐with current periodontitis with periodontal pocketing ≥ 5 mm.
‐with non‐physiological tooth mobility.

All participants completed a medical questionnaire. A necessary concomitant medication or therapy was permitted as long as it was not in the exclusion criteria. All changes in health and use of medication during the study were documented.

### 2.4 Study Products

Test product 1:
  This dentifrice was the test and experimental one. It contained 0.01% Glycyrrhiza inflate root extract; 0.1% tetrahydrocurcumin (THC), 1400 ppm F (NaF), GABA International AG.

Control product 2:
  This was the control dentifrice. It contained 1.400 ppm fluoride from sodium fluoride, GABA International AG.

Other ingredients in both products were as follows (qualitative): aqua, sorbitol, hydrated silica, hydroxyethylcellulose, PEG‐40 hydrogenated castor oil, cocamidopropyl betaine, titanium dioxide, aroma, sodium citrate, disodium lauryl sulfosuccinate, sodium fluoride, alumina, saccharin, methylparaben, tocopherol, polyaminopropyl biguanide, propylparaben, phenoxyethanol, benzoic acid, dehydroacetic acid and stearic acid. Both products were packed in identical tubes.

### 2.5 Randomization

Allocation to the test and control group was assigned by the means of a sealed opaque envelope containing a code derived from a computer‐generated randomized list. The randomization list was limited to the persons of the sponsor responsible for creation of the randomization list, preparation of the random code envelopes and preparation of the study products until final examination of the last participant and completion of the case report forms. Copies of the randomization list were kept in sealed envelopes in case of emergency that would require knowledge of the specific treatment.

### 2.6 Clinical assessments

Throughout the study, all examinations were performed by the same trained examiner (NAMR) under the same conditions at the dental faculty, ACTA Amsterdam. The examiner was blinded to the treatment randomization. At every examination, data were recorded on a case record form (CRF). Records of earlier examinations were not available at the time of re‐examinations.

The indices were scored in two randomly chosen contralateral quadrants^26^ of the mouth, either quadrants I and III or quadrants II and IV. (That is, either upper right and lower left quadrants, or upper left and lower right). Once chosen, the selected quadrants stayed the same throughout the study for each individual participant.

#### 2.6.1 Primary study parameter

Bleeding Index (BI) as Bleeding on Marginal Probing (BOMP; acc. to van der Weijden et al.^27^ 1994). The absence or presence of bleeding was scored within 30 s of probing, on a scale from 0 to 2 (0 = no bleeding, 1 = pinprick bleeding, 2 = excess bleeding). The gingival margin was probed at an angle of approximately 60° to the longitudinal axis of the tooth.

#### 2.6.2 Secondary study parameters

Modified Gingival Index (GI; visual aspect only; Lobene et al. 1986).^28^ The gingival condition was assessed using visual signs of inflammation as scored on a scale from 0 to 4 (0 = pale pink, 4 = reddish‐blue and enlarged).

Plaque Index (PI). Plaque was assessed after disclosing with Mira‐2‐Ton® (Hager & Werken GmbH & Co. KG. Duisburg, Germany) according to the Quigley & Hein plaque index,^29^ as described in detail by Paraskevas et al. (2007).^30^ The results were scored on a scale of 0 to 5.

### 2.7 Study design

The study was designed as a single‐centre, randomized, parallel group, placebo controlled and consisted of two phases, a pre‐experimental phase of three weeks and an experimental phase of four months. The participants were blinded to the product, and the examiner was blinded to treatment randomization. Text messages (short message service, SMS) were sent to remind each participant before the visits concerning the study procedures and appointments.

#### 2.7.1 Pre‐experimental phase

At the start of the pre‐experimental phase, participants were instructed to brush their teeth 2 to 3 hours prior to their appointment to avoid the risk of increased bleeding from tooth brushing.^31^, ^32^ Clinical parameters of BI, GI and PI were assessed.

Participants received professional and written instructions in the use of a manual toothbrush (Aronal® öko‐dent soft, GABA International AG) according to the Bass‐technique.^33^ Furthermore, a combination of Bocasan® (Oral‐B Laboratories, Cincinnati, OH, USA) and chlorhexidine 0.20% (Corsodyl®, GSK, Zeist, The Netherlands) was used to rinse twice daily for 1 minute during the three weeks of the pre‐experimental phase before the experimental period.^20^ The purpose of the pre‐experimental phase was to motivate participants to follow an oral hygiene regime capable of achieving and maintaining healthy gingivae. As a check for compliance, participants were asked to register the time of using the products onto a calendar record chart. During the pre‐experimental phase, the participants brushed with Everclean® dentifrice (HEMA, Amsterdam, The Netherlands) and a standard toothbrush. Participants also received a stopwatch as a timer to control their total brushing time of 2 minutes.

The next appointment for the baseline assessment (Bleeding on Marginal Probing, Modified Gingival Index, Plaque Index,) was scheduled 3 weeks later, and participants were instructed to return all remaining products received for the pre‐experimental phase toothbrush to ensure no further use of these products.

#### 2.7.2 Experimental phase

At the start of the experimental phase (baseline), participants were instructed to brush their teeth 2 to 3 hours prior to their appointment at the clinic in order to avoid the risk of increased bleeding as a result of toothbrushing.^31^, ^32^ Each participant was asked about any changes in medication, their general health, participation in other research and dental treatment received other than in the study protocol.

All three parameters (BI, GI and PI) were assessed. To ensure that participants would enter the experimental phase of the study with equally clean teeth, a dental hygienist provided a professional dental scale and polish after the clinical assessment, spending up to a maximum of 30 min. Participants were then randomly assigned to one of the two groups, test and control. New study products were provided to last them until the next appointment, including new toothbrushes (Aronal® öko‐dent soft).

All participants were instructed to brush their teeth with their assigned dentifrice for 2 minutes twice daily, using the timer. No further oral hygiene instruction was given at any stage during the remainder of the study. Participants were told to refrain from using mouthrinses, but there was no other interference with their habitual interdental oral hygiene habits.

After two months, participants were provided with a fresh exchangeable brush head for the manual toothbrush. At the end of the experimental phase (4 months), participants were instructed to brush their teeth two to three hours before their appointment at the clinic to avoid the risk of increased bleeding from tooth brushing.^31^, ^32^ Participants returned all their study products, dentifrice tubes were weighed. Clinical examinations were performed. All parameters assessed at the start of the study were re‐evaluated at this visit (BI, GI and PI).

### 2.8 Questionnaire

After completing the clinical assessment, participants were asked to complete a short questionnaire to assess their attitude to the assigned dentifrice. A visual analogue scale (VAS) was used in most questions to assess their opinions.^34^ Participants were requested to mark a point on a 10‐cm uncalibrated line, with the two end points annotating extreme responses. The left side was an extreme negative response, and the right was the positive extreme. After the trial, participants resumed using their normal oral hygiene procedures.

### 2.9 Monitoring of compliance and adverse events

The dentifrice tubes were weighed in advance and after being returned to the clinic to assess compliance. If a participant discontinued the study, their reasons and circumstances of discontinuation were documented. Any adverse events reported by participants during the course of the study were appropriately recorded.

### 2.10 Statistical analysis

Computations were performed using R (https://www.r‐project.org).^35^The BI scores were used as the main response variable. Analyses comparing differences between the test and control groups were performed using Mann‐Whitney tests. Wilcoxon tests were performed to analyse differences within the groups between sessions. An analysis of covariance (ANCOVA) was performed with the scores of session 1 as the covariate. *p*‐values ≤ 0.05 were accepted as statistically significant. Differences in mean plaque score reduction between dentifrices were expressed as a ratio and as a percentage reduction relative to the control dentifrice.^36^ The statistical analysis was performed blinded to product allocation.

## 3 RESULTS

**FIGURE 1:**
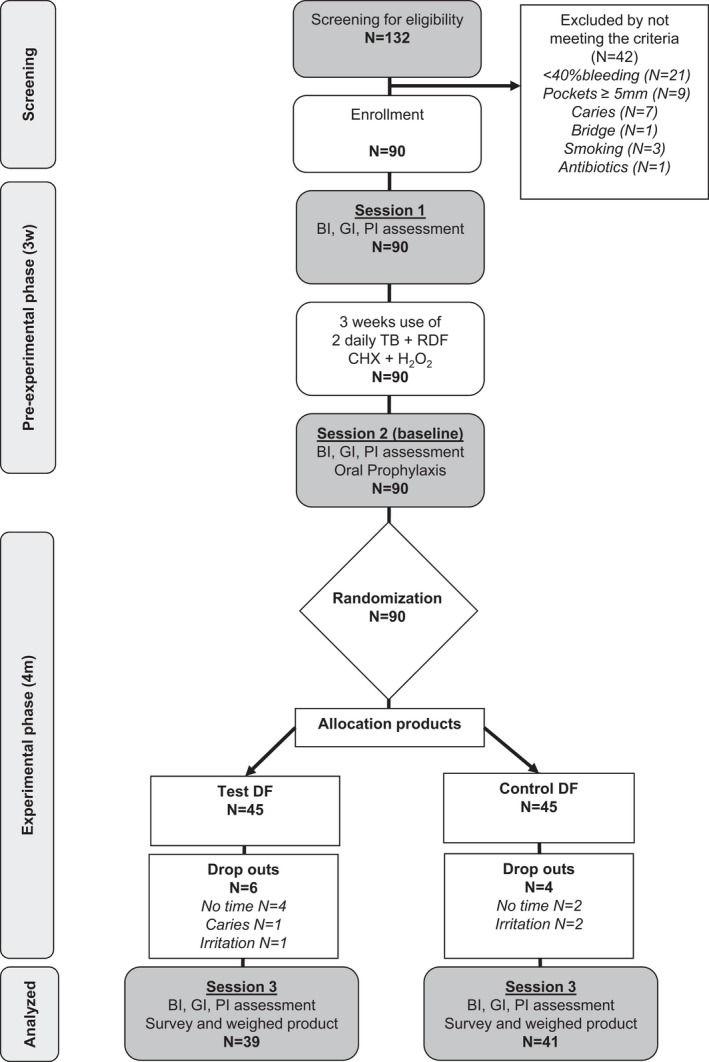
Flow chart of the study design and enrollment of the study population

The study flow proposed by Consort,^21^ is shown in Figure 1, which also provides details about dropouts of the study. A total of 132 possible participants were recruited, of whom 90 participants were found to be eligible.

In total, 80 participants completed the protocol. Table 1 shows their general characteristics. About 80% of participants were female, and the mean age of all participants was 22.2 years (SD 2.50) with a range of 18–29 years. There was no statistically significant difference between the groups in relation to age (*p* = 0.07) and gender (*p* = 0.66).

**TABLE 1:** Study demographic characteristics

		Test group N = 39	Control group N = 41	*p*‐value (between groups)
Gender				
Female	n (%)	32 (82%)	32 (78%)	0.655^a^
Male	*n* (%)	7 (18%)	9 (22%)	
Age (years)	Mean (SD)	21.64 (2.15)	22.66 (2.74)	0.069^b^
Range (years)	min‐max	18–26	18–29	

^a^ Chi‐square test

^b^ T test

Table 2 shows the mean scores of both groups, for all indices and at all sessions.

**TABLE 2:** (a) Mean bleeding scores (BOMP) for both groups at all sessions. (b) Mean gingivitis scores (MGI) for both groups at all sessions. (c) Mean plaque scores (Q&H) for both groups at all sessions.

a) Bleeding (BOMP)	N	Session 1 (pre‐experimental)	Session 2 (baseline)	Session 3 (end)	Diff (Session 1–2) MDiff (SD); *p*‐value^b^; % diff.^d^	Diff (Session 2–3) MDiff (SD); *p*‐value^b^; % diff.^d^
Group Test	39	1.11 (0.23)	0.42 (0.18)	0.52 (0.25)	−0.68 (0.22); <0.001; −62.2%	0.10 (0.26); 0.08; 23.8%
Group Control	41	1.13 (0.22)	0.43 (0.18)	0.56 (0.25)	−0.69 (0.23); <0.001; −61.9%	0.13 (0.19); <0.001; 30.2%
*p*‐values analysis between groups		0.44^a^	0.83^a^; 0.97^c^	0.44^a^; 0.52^c^	0.57^a^; 0.97^c^	0.15^a^; 0.55^c^
95% CI		−0.12; 0.07	−009; 0.07	−0.15; 0.07	−0.08; 0.13	−0.16; 0.03
Ratio; % diff.^d^					0.99x^d^; −1.43%^d^	0.77x^d^; −23.1%^d^

b) Gingivitis (MGI)	N	Session 1 (pre‐experimental)	Session 2 (baseline)	Session 3 (end)	Diff (Session 1–2) MDiff (SD); *p*‐value^b^; % diff.^d^	Diff (Session 2–3) MDiff (SD); *p*‐value^b^; % diff.^d^
Group Test	39	0.61 (0.38)	0.23 (0.12)	0.27 (0.17)	−0.38 (0.31); <0.001; −62.3%	0.04 (0.14); 0.12; 17.4%
Group Control	41	0.63 (0.26)	0.27 (0.11)	0.31 (0.16)	−0.36 (0.23); <0.001; −57.1%	0.03 (0.12); 0.09; 14.8%
*p*‐values analysis between groups		0.32^a^	0.11^a^; 0.09 ^c^	0.26^a^; 0.35^c^	0.80^a^; 0.09^c^	0.87^a^; 0.79^c^
95% CI		−0.18; 0.06	−0.09; 0.01	−0.10; 0.02	−0.10; 0.12	−0.06; 0.07
Ratio; % diff.^d^					1.06x^d^; 5.6%^d^	1.0x^d^; 0%^d^

c) Plaque (Q&H)	N	Session 1 (pre‐experimental)	Session 2 (baseline)	Session 3 (end)	Diff (Session 1–2) MDiff (SD); *p*‐value^b^; % diff.^d^	Diff (Session 2–3) Diff (SD); *p*‐value^b^; % diff.^d^
*Group Test*	39	1.84 (0.42)	0.76 (0.62)	1.89 (0.46)	−1.08 (0.54); <0.001; −58.7%	1.12 (0.54); <0.001; 148.7%
*Group Control*	41	1.91 (0.40)	0.80 (0.50)	1.98 (0.43)	−1.11 (0.46); <0.001; −58.1%	1.18 (0.47); <0.001; 147.5%
*p‐values analysis between groups*		0.35^a^	0.40^a^; 0.93^c^	0.33^a^; 0.53^c^	0.75^a^; 0.93^c^	0.99^a^; 0.58^c^
*95% CI*		−0.26; 0.10	−0.26; 0.12	−0.29; 0.12	−0.18; 0.25	−0.23; 0.20
*Ratio*; % *diff*.^d^					0.97x^d^; −2.70%^d^	0.96x^d^; −4.24%^d^

Note

*Session 1* = pre‐experimental phase (3 weeks), *Session 2* = baseline, *Session 3* = end (4 months). Standard deviation in parentheses. Ratio = (test dentifrice mean plaque reduction)/ (reference dentifrice mean plaque reduction). % difference in score reduction = 100% × (test dentifrice mean plaque reduction‐reference dentifrice mean plaque reduction)/reference mean score reduction^36^

^a^ from Mann‐Whitney test.

^b^ *p*‐value from Wilcoxon test between sessions within each group.

^c^ *p*‐value from ANCOVA analyses between groups with Session 1 as covariate.

^d^ Difference in reduction mean scores between dentifrices is expressed as a ratio and percentage of reduction score for the reference dentifrice. A positive value indicates greater plaque reduction in favour of brushing with the test dentifrice as compared to the reference dentifrice.

At the first assessment (pre‐experimental phase), the mean scores for all indices did not differ between the groups. At the second assessment (baseline), none of the values for any of the parameters of interest showed significant differences between groups.

At the third session, which was held after four months, the values of BI, GI and PI did not show any significant differences between the groups. No statistically significant differences (*p* < 0.05) were observed between the groups at any stage of the study, for any of the parameters or at the different measurement times. However, for both groups, all three parameters decreased significantly (*p* < 0.001) during the pre‐experimental period—that is, between the first assessment and the baseline measurements.

Results from the questionnaire showed that a statistically significant difference (*p* = 0.003) could be observed regarding the strongness of the taste between the two dentifrices. Participants expressed their preference for the control dentifrice (Table 3).

**TABLE 3:** Questionnaire related to the attitude of the participants to the assigned dentifrice using a Visual Analogue Scale (VAS) presented as mean and standard deviation (SD) with negative extremes on the left and positive extremes on the right (from 0 to 10)

Question	Extreme		Dentifrice		
	From	To	TEST N = 39 Mean (SD)	CONTROL N = 41 Mean (SD)	*p* ^a^
What is your opinion of the taste of the dentifrice?	very bad	very nice	5.26 (2.10)	5.66 (2.14)	0.405
What is your opinion of the freshness of the dentifrice?	not fresh	very fresh	4.63 (2.11)	5.12 (1.85)	0.268
Do you consider this	not fresh at all	too fresh	3.40 (1.55)	3.81 (1.28)	0.195
What is your opinion of the strongness of the taste of the dentifrice?	not strong at all	too strong	3.25 (1.51)	4.30 (1.53)	0.003
If yes, for how long does this fresh/clean feeling persist after brushing?	very short	very long	4.03 (1.64)	3.57 (1.36)	0.191
			32(Yes)/7(No)	39(Yes)/1(No)	0.057^b^
What is your opinion of the taste of food and drinks after brushing?	changed negatively	changed positively	4.71 (1.23)	4.51 (1.36)	0.500
What is your opinion of the foaming effect of the dentifrice?	does not foam	too much foam	4.76 (1.41)	4.45 (1.59)	0.360
Would you use this dentifrice in the future?	absolutely not	for sure	4.07 (2.64)	4.44 (2.52)	0.529
Do you consider this dentifrice is improving your gums?	changed negatively	changed positively	4.53 (1.70)	4.98 (2.00)	0.288
What is your opinion about the brush?	very bad	very nice	5.78 (2.17)	6.05 (2.15)	0.579
What is your opinion about the stiffness of the filaments of the brush?	too soft	too stiff	4.27 (1.61)	4.25 (1.46)	0.958
Do you have the feeling that the brush cleans well?	does not clean at all	cleans very well	5.69 (2.13)	5.44 (2.07)	0.598

^a^ T test.

^b^ Chi‐square statistic with continuity correction.

Weighing of the dentifrice tubes indicated an average use of 226.33 g (SD 85.6) across the 78 participants during the four months of the study. Divided by groups, this resulted in a mean of 209.5 g (66.7) for the test group, whereas for the control group the mean was 241.6 g (98.0). This difference was not statistically significant (*p* = 0.093).

### 3.1 Adverse events

In the pre‐experimental phase, eight participants complained about a burning sensation, alteration in taste and irritation of mucosa. Three of these eight participants stopped using the Bocasan mouthrinse. During the experimental phase, two adverse events in the control group were registered: one person dropped out after complaining of irritation of the gingiva because of the use of the prescribed toothbrush; another dropped out because of irritation of the mouth, with ulceration, bad taste, and warm and sour sensitivity. One adverse event registered for the test group was a dropout who complained about a burning feeling in the mouth. See Figure 1.

## 4 DISCUSSION

The design of the present study was based on a model published by Svatun et al.^18^, ^37^ The current study tested after gingival health was established through a prophylactic aid, the concept whether a dentifrice can prevent deterioration of the gingival status. It is presumed that without the ongoing use of the prophylactic aid, improved gingival health tends to fade away over time and returns to its original values. It has been observed that the most marked deterioration occurs within the first three months following the pre‐experimental phase, indicating a relatively rapid loss of the dedication required to maintain effective plaque control.^6^ In both the Svatun studies,^18^, ^37^ a moderately inflamed gingival condition in a group of young, health‐conscious volunteers was brought to an excellent state of health by professional cleaning and oral hygiene instruction. This study model proved to be effective in testing dentifrice to suppress both plaque accumulation and the development of gingivitis.

In the present study, the Svatun^18^ model was adapted so that during the three‐week pre‐experimental phase, oral hygiene instruction was combined with the use of oxygenating and chlorhexidine (CHX) rinse. The use of these mouthrinses was added to enable participants to enter the experimental phase with the healthiest possible gingival condition. This would assist the researchers in discerning the maximum differences in gingivitis levels between Day 0 and baseline. The same adaptation was used previously and was effective for reducing the mean score of an inflammatory parameter.^4^

From a systematic review, there is moderate evidence that a combination of CHX and an oxygenating rinse (H_2_O_2_) reduces tooth staining without interfering with the plaque growth inhibition of CHX.^38^ This might be why no participants dropped out during the pre‐experimental phase due to CHX staining. One of the most widely used detergents in dentifrice is sodium lauryl sulphate (SLS). There has been a lengthy discussion regarding whether SLS and CHX counteract each other. To date, the general recommendation by CHX manufacturers is to rinse with CHX 30 min after brushing or to use an SLS‐free dentifrice. This is in contrast to the findings of a systematic review that showed that the combination of CHX and a SLS dentifrice is not contraindicated.^39^ Therefore, the regular dentifrice in the pre‐experimental phase could be used without any specific instruction regarding CHX effectivity.

At session 1 (pre‐experimental), no significant differences between groups were observed, indicating that the groups were comparable. Again, at session 2 (baseline), no significant differences were observed. However, as expected, significant decreases in all measured variables were noted after the participants received the instructions and both mouth rinses. Although no statistically significant differences between groups were observed at session 3, there might have been an effect of the test dentifrice; if so, it was not large enough to be detected with the statistical method for analysing a two‐group parallel design.

A Wilcoxon test to check for differences within groups over time indicated a significant increase for the control group regarding mean BOMP scores. The test group, however, did not exhibit a significant increase. See Table 2. As we expected based on the Svatun et al.^18^ paper the bleeding scores in the control group did not return to approximately the baseline level. This in contrast to the PI. Why this is the case cannot be explained based on the present findings. We have reported what we observed from the analysis of the collected data. Fortunately, this was a controlled study so that both groups were affected by any effect that may have resulted in the present findings.

In this study, licorice and turmeric root (also known as glycyrrhiza glabra and curcuma xanthorrhiza respectively) were the active ingredient in the dentifrice under investigation. Both additives were employed medicinally by ancient civilizations such as the Romans and Chinese. For many years, the capacities of these plants have been used to improve human health.^40^

Licorice is also used as an important sweetening and flavouring agent in food products, beverages, medicines and dentifrice.^41^, ^42^ In modern medicine, isolated components of licorice have been shown to inhibit the growth and cytopathology of viruses—such as hepatitis A^43^ and C,^44^, ^45^ herpes zoster,^46^ HIV,^47^, ^48^ herpes simplex,^49^, ^50^ and cytomegalovirus.^51^ Evidence for a therapeutic application of licorice in oral diseases has also been reported.^12^, ^42^, ^52^ Similarly, curcuma is widely investigated, and numerous studies have reported medicinal benefits from its components, the curcuminoids.^53^, ^54^

Although both licorice and turmeric are often suggested to be effective in oral care products, the proposed mechanism is hardly ever mentioned. It has also been reported that the mechanism by which licorice may inhibit dental plaque formation is not fully understood.^14^ Licorice however has shown to have antibacterial activity against S.mutans^15^, ^16^ and also anti‐adhesive properties against P.gingivalis.^55^ Curcumin is most commonly reported as an antioxidant with antibacterial and anti‐inflammatory effects.^54^, ^56^, ^57^

The combination of licorice and turmeric is well known in food, tea and skin products. It is presumed that it may also be a promising adjective for dentifrice products. This combination promotes strong anti‐oxidative activity, which supports the action of preventing gingivitis. Before the trial, the efficacy of an enriched licorice and turmeric root extract was investigated in vitro. The model used is well established for testing anti‐inflammatory effects of compounds as it examines various inflammatory mediators.^58^, ^59^ Parameters such as PGE2 and IL‐1 are known to play a crucial role in gingivitis. The in vitro results showed that the tested extracts potently prevented both PGE2 and IL‐1 release. However, the statistical comparison between groups at each stage of the in vivo study, for all parameters, showed no significant differences.

The reason for the incremental differences between sessions not being reflected in the parallel analyses between groups is unclear and requires speculation. Apparently, the effect is detectable but small. Maybe a pre‐trial phase without the use of both mouthrinses would have made difference between both test and control groups in time clearer. It is conceivable that the effect of the pre‐trial phase might have lasted throughout the four‐month session. However, Van Leeuwen et al.^60^ demonstrated limited residual effects from a rinsing treatment after four months.

In addition, the professional cleaning and polishing likely contributed to the improved oral health scores. Both of these interventions have a known short‐term effect on all parameters.^20^, ^61^, ^62^ This gives researchers the opportunity to observe an effect of a participant returning to their habitual level for the parameters assessed at the end of the trial. The process was described by Svatun *et al*.^18^ The combination of oral hygiene instruction, professional prophylaxis, and the use of the combination of both CHX and the oxygenating rinse may have resulted in an overwhelming effect, which would limit the potential effect of the test dentifrice. Other possible explanations for the lack of adjuvant efficiency may have been insufficient concentrations of the natural ingredients and/or chemical incompatibility in the dentifrice formulation, which also contains a mixture of anionic detergents and organic antibacterial surface agent, which may have counteracted with the presumed active ingredients.^63^

## 5 CONCLUSION

Within the limits of the current study design, dentifrice formulation and concentration of turmeric/licorice extracts the results show that the adjuvant effect of the natural ingredients in the test dentifrice was not evident on clinical parameters of gingivitis and plaque. A modified set up of protocol, formulation and concentration could be subject to future research.

## 6 CLINICAL RELEVANCE

### 6.1 Scientific rationale for the study

When new oral hygiene products are developed, it is of important to assess their effectiveness and safety. Such studies can inform dental professionals regarding a dentifrice's inhibitory effects on plaque and its effect on gingivitis parameters.

### 6.2 Principal findings

After four months of using the test dentifrice with a specific formulation and concentration of turmeric and licorice, no adjuvant effect of these medicinal herbal extracts was found in comparison with the control dentifrice for the employed study design.

### 6.3 Practical implications

A practical implication of the study is that a design with a pre‐experimental phase in which a combination of both CHX and an oxygenating agent as prophylactic aid is used could be of influence to detect differences in four‐month studies.

## CONFLICT OF INTEREST

The study was performed on commission from ACTA Dental Research BV (ADR). ADR received financial support from GABA International AG, Switzerland. ADR appointed this project to the Department of Periodontology at ACTA. Furthermore, Van der Weijden, Slot and Rosema have previously received either external advisor fees, lecturer fees or research grants from toothbrush and dentifrice manufacturers. These manufacturers included Colgate, Dentaid, GABA, Lactona, GSK, Oral‐B, Procter & Gamble, Sara Lee, Sunstar and Unilever. Hoenderdos, Versteeg and Valkenburg report no conflict of interests.

## AUTHOR CONTRIBUTIONS

CV: contributed to analysis and interpretation, drafted the manuscript. NAMR: contributed to conception and design, collected the data, contributed to analysis and critically revised the manuscript. NLHH: contributed to the coordination and execution of the project and critically revised the manuscript. PAV: contributed to execution of the project, ethical approval procedure and critically revised the manuscript. DES: contributed to conception and design, analysis and interpretation, and critically revised the manuscript. GAW: contributed to conception and design, analysis and interpretation, and critically revised the manuscript. All authors: Gave final approval and agreed to be accountable for all aspects of work ensuring integrity and accuracy.

## Data Availability

The data that support the findings of this study are available from the corresponding author upon reasonable request.
